# 'Offensive' snakes: cultural beliefs and practices related to snakebites in a Brazilian rural settlement

**DOI:** 10.1186/1746-4269-6-13

**Published:** 2010-03-26

**Authors:** Dídac S Fita, Eraldo M Costa Neto, Alexandre Schiavetti

**Affiliations:** 1 Department of Biodiversity Conservation, El Colegio de la Frontera Sur (ECOSUR), C.P. 29290, Apto Postal 63, San Cristóbal de las Casas, Chiapas, México; 2 Departament of Biology, Universidade Estadual de Feira de Santana, 44036-900, Feira de Santana, Bahia, Brazil; 3 Departament of Agrarian and Environment Sciences, Universidade Estadual de Santa Cruz, 45662-900, Ilhéus, Bahia, Brazil

## Abstract

This paper records the meaning of the term 'offense' and the folk knowledge related to local beliefs and practices of folk medicine that prevent and treat snake bites, as well as the implications for the conservation of snakes in the county of Pedra Branca, Bahia State, Brazil. The data was recorded from September to November 2006 by means of open-ended interviews performed with 74 individuals of both genders, whose ages ranged from 4 to 89 years old. The results show that the local terms biting, stinging and pricking are synonymous and used as equivalent to offending. All these terms mean to attack. A total of 23 types of 'snakes' were recorded, based on their local names. Four of them are Viperidae, which were considered the most dangerous to humans, besides causing more aversion and fear in the population. In general, local people have strong negative behavior towards snakes, killing them whenever possible. Until the antivenom was present and available, the locals used only charms, prayers and homemade remedies to treat or protect themselves and others from snake bites. Nowadays, people do not pay attention to these things because, basically, the antivenom is now easily obtained at regional hospitals. It is understood that the ethnozoological knowledge, customs and popular practices of the Pedra Branca inhabitants result in a valuable cultural resource which should be considered in every discussion regarding public health, sanitation and practices of traditional medicine, as well as in faunistic studies and conservation strategies for local biological diversity.

## Introduction

Snakes are among the animals that have most influenced the human psyche since ancient times. Images (cultural representations), myths and beliefs regarding these reptiles are present in many societies due to their culturally salient biological and behavioral characteristics, such as their speed and agility, the bifid tongue, unblinking lidless eyes, their ability to periodically renew their skin, their mode of mating, and the ability to produce and inject a deadly substance [1]. In ancient times, many people worshiped snakes to try to appease them in order to avoid the evil they could cause. Attributes such as wisdom, cunning, power, fertility, sexuality and renewal of life have been (and, to an extent, still are) attributed to them by different people in India, Egypt, ancient Europe, ancient Persia, Mexico and much of Africa [2]. Even in regions where snakes were not objects of worship, they were used as symbols, amulets and religious elements [3].

In Mexico, the Aztecs made extensive use of the ophidiofauna: several species were consumed, offered to the gods and used as medicines [4]. For the Mayans, the snake had a religious significance as a sacred animal force, bound to various divine figures, representing the union of opposing forces in nature. For example, to the Tsotsil Mayans from the municipality of Zinacantán, state of Chiapas, snakes are the earth lord's daughters [4]. In Central America, as well as several other parts of the world, snakes play an important role in religious thought and in the day-to-day life of people, representing life cycles depending on the attributes and contexts in which they are found. The serpent is the beginning and the end, water and fire, movement, life and death [5].

Although snakes are considered as sacred beings in some cultures, most people view them as the most abominable living creatures, a view that promotes unnecessary killing and puts sensitive food chains at risk [6]. Of the world's 3,232 species of snakes, around 500 are venomous. However, many people believe that all snakes are dangerous and aggressive. Almost half of Americans have some degree of anxiety when they see a snake, and 20% are terrified by even pictures of snakes [2]. This irrational fear, called ophidiophobia, far exceeds the actual risk of getting bitten, since of the 7,000 - 8,000 people a year bitten by venomous snakes in the United States, only about five die and 20-30% receive dry bites [2]. Whether they are venomous or not, people usually refer to snakes with anthropomorphic terms, such as ugly, repugnant, repulsive, cruel, treacherous, vicious, vindictive, offensive, etc. This biophobic reaction results from an exaggerated, superstitious and unreasonable fear, often caused by false and absurd ideas that people have about the behavioral aspects of these reptiles, which are interpreted based on non-scientific cultural patterns. The Judeo-Christian tradition and European beliefs added the devilish, harmful element [7].

In Brazil, snakes are objects of innumerable stories, legends and beliefs that are deeply rooted in popular culture [7,8]. Moreover, there is a rich body of knowledge, popular customs and practices that are related to the victim's treatment and to fighting these reptiles [9]. Because of the way they are perceived, snakes are most often associated with the image of venomous animals which are capable of causing major damage, often leading to death. Consequently, people's first reaction when encountering a snake is to exterminate it, regardless of whether it represents a valid threat or not. Thus, throughout the world there are different ways of thinking and acting when snake-caused accidents occur. At the same time, homemade remedies, prayers and charms aim to prevent or treat injuries caused by the deleterious action of venom [3,9-18].

This article will briefly discuss how the residents of the village of Pedra Branca, municipality of Santa Teresinha (Bahia, Brazil), interact with snake species. It records their folk knowledge with respect to local beliefs and practices of traditional medicine that prevent and treat ophidism, and the implications for the conservation of snakes in the region.

### Study Area

The region, known as Serra da Jibóia (literally boa's mountain), is located at the approximate coordinates 12°51' south latitude and 39°28' west longitude. Extending from the north-south direction, its crest measures 26 km long and reaches maximum elevation of about 820 m.a.s.l. This massive mountain is located at an ecotone, giving it a wide variety of climates, relief, soil, vegetation and wildlife [19].

The village of Pedra Branca is situated at the base of the Serra da Jibóia, within the municipality of Santa Terezinha. According to the local health agent, there were about 380 residents in June 2007. The total population for the entire municipality of Santa Terezinha was 9.914 inhabitants [20].

Living in a basically rural area, the population of Pedra Branca depends on the cultivation of cassava (*Manihot esculenta *Crantz, Euphorbiaceae) as the main economic activity. There is also cultivation of grapes (*Vitis *sp.) for production of homemade wine and the fruit trade. Livestock are also important, especially cattle and goats.

The village has a health center that serves the community in a limited way (due to lack of material and human resources). In cases of venomous animal-bites, which are very common in rural areas, the nearest medical facility where the individual can be treated is the Emergency Room of Santa Terezinha (up to 13 km away by paved road) or the hospital in the city of Castro Alves (up to 26 km away, 11 of which are in bad condition). These two places have antiophidic, antiscorpionic and antiarachnidic serums in stock.

## Materials and methods

Fieldwork was performed from September to November 2006, totaling 53 days of living in the village. Later, there was a stay of 15 days between June and July 2007 with the aim of giving testimony to previous interviews and to record new information, as well as to participate in some sociocultural activities of the community.

A total of 74 individuals were interviewed, namely 35 men and 39 women whose ages ranged from 4 to 89 years old. The contact with individuals of different age groups allowed for the registration of transgenerational diffusion of ethnozoological knowledge. Local inhabitants are mainly small farmers and most of them are of Afro-Brazilian origin. All of them can only speak Portuguese.

An Open and Clarified Consent Term was elaborated based on the Brazilian Health Council Resolution number 196/1996, which rules the ethical aspects of the research involving human beings. It was read to the villagers and distributed among those who participated in the study. The main objectives of the research were explained clearly at the beginning of each new interview and people were asked if they wanted to participate.

Initially, we interviewed any inhabitant about the traditional knowledge and beliefs regarding snakes. The 'snowball' technique [21] was used, as some villagers indicated others who were more knowledgeable on the surveyed subject. Interview data was recorded using a digital tape-recorder and camera following various techniques of qualitative research for ethnographic records, such as: open (free talks) and semi-structured interviews (based on a list of topics previously chosen), and *ad libitum *observation of the individuals during interviews (including their facial and body expressions). The interviews were individual and/or collective and occurred in a variety of contexts: residences, plantations, the street, grocery stores, the health center, the local school, manioc flour house, and during trips to the forest.

Collected specimens, photographs from wildlife guides [22,23], as well as pamphlets and posters showing pictures of snakes made it possible to carry out interviews stimulated by the presentation (and representation) of animals depicted in these materials, asking the respondents about the snakes they observed (local name and aspects of their biology and ecology), their impressions and attitudes towards them, and especially the local beliefs regarding snakebites.

Data were analyzed using the union model [24]. According to this model, all available information on the surveyed subject is to be considered. Controls were done both through consistency checking tests and reply validity tests, which make use of repeated inquiries in synchronic and diachronic conditions, respectively. The former occurred when the same question was put to different people soon after each other; the latter occurred when the same question was asked to the same person at different times [25]. In order to observe the similarities between ethnozoological knowledge and scientific knowledge related to snakes' life history, we constructed comparative cognition tables in which excerpts of the interviews are compared to excerpts from pertinent literature [26].

Specimens were identified by specialists and are kept at the scientific collection of the Laboratory of Vertebrate Zoology of the Universidade Estadual de Santa Cruz (UESC), state of Bahia. All ethnographic materials (recordings, transcripts, photographs, and drawings) are kept at the Laboratory of Ethnobiology and Ethnoecology of the Universidade Estadual de Feira de Santana (UEFS), state of Bahia, for evidential purposes.

## Results and Discussion

Snake-caused injuries: implications for perception, knowledge and attitudes toward these reptiles.

According to the interviewees, the local terms biting, stinging and pricking are synonymous and used as equivalent to offending. In this article, the word offense means a direct injury, real and/or imaginary, caused by any animal to a person, seriously jeopardizing (or not) his welfare and health. In addition, the word offense refers to both the animal's 'predisposition' to attack and the mechanical action of this behavior (bite, sting, and prick). Several of the interviewed subjects (n = 36) have insisted on the fact that it also means that the animal is immediately considered a threat to human beings because it carries venom, and thus may cause someone's death.

There are some references in the literature regarding the use of the term offense as equivalent (and substitute) of the words sting and bite. In his book 'Zoobiblion', Zacharias Wagener, who gathered information from 1634 to 1641 on the Brazilian fauna, wrote: 'In the case of a woman being bitten, or in any way offended by this pernicious animal (referring to lizards), she does not suffer the least harm; but if a single drop of blood is only sucked, she is doomed to die' [7]. Another example can be extracted from Lenko [27], who commented that in 1727 the Portuguese physician John Curvo Semedo, speaking on the use of the rattle of the rattlesnake as folk medicine, wrote: ' [...] it is widely used [...] in cases of bites from snakes. [...] they do it in several ways: the rattle is to be ground and a tea is made, which is drunk by the person offended by a snake [...]'. In Alto Juruá (Acre State), Souza et al. [28] recorded the testimony of a rubber tapper: ' [...] St. Benedict is the protector against snakes, and the person who has devotion to him is not offended. The person should think of St. Benedict before the snake bites [...]'. In the state of Bahia, Costa-Neto [29], Costa-Neto & Magalhães [30] and Silva & Costa-Neto [31] also documented the use of the term offense as a synonym for snake and insect bites and stings. Perhaps the idea that snakes sting is due to the great speed with which most of them bite and release, then producing the sensation of having received a sting [32].

Amongst the inhabitants of Pedra Branca and nearby settlements, the offense could be perceived as one of the main aspects used in the cognitive formation of the 'Insect' ethnotaxon. This does not mean that the act of offending is an exclusive characteristic of the representatives of this ethnozoological semantic domain, but it is quite relevant when the word 'insect' itself is associated to every organism culturally considered as bad, harmful and dangerous, especially for human health: *When we least expect the snake bites, offends us [...]. What do we call that? Is it not an insect? It is! *(Mrs. N., 57 years old). That is why snakes are perceived and classified as 'insects'.

A total of 23 types of 'snakes' were recorded, based on their local names (Table 1). Four of them belong to the family Viperidae: surucuru-pico-de-jaca or bushmaster (*Lachesis muta*), jararaca-malha-de-sapo or whitetail lancehead (*Bothrops leucurus*), jararaca-cabo-branco (*B. leucurus*), and cascavel or rattlesnake (*Crotalus durissus cascavella*). These snakes were considered the most dangerous to humans, besides causing more aversion and fear in the population. Of the 31 respondents who commented on the jararaca-cabo-branco, only one said that it is actually the offspring of the jararaca-malha-de-sapo (*B. leucurus*) and not a different species as generally believed by local people. Precisely, Viperidae comprises those species having solenoglyphous dentition (hollow fangs that are located at the front of the maxilla) for venom inoculation, being of great medical interest [1]. Only four interviewees have cited the fangs that some snakes possess to inoculate the venom: *A small bag where it has the poison, in the tooth *(F., 28 years).

**Table 1:** Limbless squamate species referred to as dangerous to human beings according to the 74 dwellers of Pedra Branca village, Bahia State, Brazil.

Species recorded	Local names (Portuguese and English)	N° of citation	Is it poisonous?	
			
			Yes	No
Amphisbaenidae				
*Amphisbaena *sp.	Cobra-de-duas-cabeças, cobra-cega Two-headed snakes, worm lizard	12	83,3	16,7
Boidae				
*Boa constrictor constrictor *(Linnaeus, 1758)	Jibóia Boa	7		100
Viperidae				
*Lachesis muta *(Linnaeus, 1766)	Surucucu-pico-de-jaca, surucuru-malha-de-fogo Bushmaster, whitetail lancehead	45	100	
*Bothrops leucurus *(Wagler, 1824)	Jararaca-malha-de-sapo, jararaca-quatro-ventas Whitetail lancehead	41	100	
*Crotalus durissus cascavella *(Wagler, 1824)	Cascavel Rattlesnake	40	100	
*Bothrops leucurus *(Wagler, 1824)	Jararaca-cabo-branco Whitetail lancehead	31	100	
*Bothriopsis bilineata bilineata *(Wied, 1825)	Surucucu-de-pindoba, pingo-de-ouro Two-striped forest pitviper	8	100	
Colubridae				
*Boiruna sertaneja *Zaher, 1996	Buiúna Black snake	35	43	57
*Drymarchon corais *(Boié, 1827)	Papa-pinto Índigo snake	25	60	40
*Chironius *sp., *Leptophis *sp., *Philodryas *sp., *Oxybelis aeneus *(Wagler, 1824)	Cobra-espada Parrot snake, rain frog snake	25	8	92
*Chironius *sp., *Leptophis *sp., *Philodryas *sp., *Oxybelis aeneus *(Wagler, 1824)	Cobra-de-cipó Rain frog snake, parrot snake	24	4,2	95,8
*Oxyrhopus trigeminus *Dúmeril, Bibron and Duméril, 1854	Cobra-coral False coral snake	22	100	
*Micrurus *sp.	Cobra-coral True coral snake			
*Spilotes pullatus *(Linnaeus, 1758)	Cainana Tiger rat snake	19	10,5	89,5
*Helicops *sp., *Liophis *sp.	Cobra-d'água Water snake	10	20	80
*Waglerophis merremii *(Wagler, 1824)	Esparradeira, jararaca-esparra Flat snake	5	80	20
*Bothrops *sp.	Jaracuçu Viper	5	20	80
*Liophis viridis *Günther, 1862, *Philodryas olfersii *(Lichtenstein, 1823)	Cobra-verde Green snake	4		100
*Tantilla melanocephala *(Linnaeus, 1758)	Correia-de-veado Black-headed snake	2		100
*Mastigodryas bifossatus *(Raddi, 1820)	Malha-de-traíra Racer snake	2		100
*Tantilla melanocephala *(Linnaeus, 1758) *Pseudoboa nigra *(Duméril, Bibron and Duméril, 1854)	Cobra-cinco-horas, cobra-seis-horas Black-headed snake False boa	2		100
*Liophis *sp., *Thamnodynastes *sp.	Jaracaquinha Striped swamp snake	1		100
*Sibynomorphus neuwiedii *(Ihering, 1910)	Dormideira Neuwied's tree snake	4		

Snakes belonging to the family Colubridae, such as buiúna or black snake (*Boiruna sertaneja*), papa-pinto or indigo snake (*Drymarchon corais*), cobra-espada or parrot snake (*Leptophis *sp., *Philodryas *sp.), cobra-cipó or rain frog snake (*Leptophis *sp., *Philodryas *sp.), caninana or tiger rat snake (*Spilotes pullatus*), and cobra-d'água or water snake (*Helicops *sp.), were also cited. Interviewees were hesitant to say if these snakes were poisonous or not. According to Cardoso et al. [1], although they are not considered venomous snakes, in some cases their opisthoglyph dentition (enlarged fangs on the rear of the maxilla) allow them to inoculate some amount of toxin and this eventually cause a notorious local inflammation. These authors emphasize that the genera *Boiruna *and *Philodryas *may cause accidents with local pattern similar to that caused by *Bothrops *and *Lachesis*, that is, with proteolytic (acute inflammatory), coagulant and hemorrhagic actions.

*Boiruna sertaneja *and *D. corais *are examples of taxa about which there are divergent opinions regarding the alleged presence of venom. Of the respondents, 15 commented that these snakes are 'poisonous' only during the time of the year they reproduce, corresponding to June to August. It is worth mentioning that *D. corais*, due to its aglyphous teeth, cannot be considered as venomous under any circumstances [22].

Of the 22 interviewees that mentioned coral snakes, only two reported differences between the false coral snake (*Oxyrhopus *sp.) and the true coral snake (*Micrurus *sp.). However, both these snakes were always grouped together with the viperid snakes as being very dangerous and poisonous.

The other snakes, including the boa (*Boa constrictor constrictor*), received low citation, having in common the fact of being treated as not venomous, although they can offend someone through their bites. It is believed that the rain frog snake offends by hiting someone with its own body (see Table 2). This fact suggests that, in the village of Pedra Branca, the question of being offended by snakes is not limited to the presence or absence of the 'poison', but the mechanical action itself, even if this does not involve biting and/or stinging.

**Table 2:** Beliefs related directly or indirectly to the injuries (real and/or imaginary) caused by snake bites, according to the dwellers of the county of Pedra Branca, Santa Terezinha, Bahia State, Brazil.

Beliefs	Snake species involved	Number of interviewees who	
		
		Believe	Do not believe
If you don't kill the snake that has bitten you, it stays spinning on the ground and this strengths the action of the injected poison.*	Snakes in general	11	7
If the snake sees you (in the woods) but you don't see it, you fall ill (swollen ganglia, fever etc.).	Snakes in general	4	9
Snakes cause swelling of the ganglia.	Snakes in general	3	
Snakes do not like pregnant women. They are angry with them. They will go directly toward them to beat them with their tail.	Snakes in general	15	3
If you strike a match on the wound, it draws the poison, increasing the flame.	Snakes in general		3
If the individual does not beat on the snake's head (to kill it), the devil will heal it.*	Rain frog snakes		2
If someone beats the snake without killing it, the snake goes to that person's house and stays on the roof. Then the person falls ill and dies. When the coffin leaves, the snakes go after it to check.*	Rain frog snakes		3
It is not poisonous, but if someone is bitten he will be drained [faded out] in the same way the snake is.	Rain frog snakes	9	
It does not bite, but one weakens if once bitten by it.	Rain frog snakes	6	
If someone beats the snake without killing it, the snake lies in ambush waiting for this (or another person) pass again to attack.*	Parrot snakes Tiger rat snakes	15	4
After the snake has bitten someone, it goes to the roof to wait for the result of the bite.	Tiger rat snakes	2	3
Because of its poison a venomous snake continues to move, even after being killed.	Snakes in general	6	
The tail, once cut, moves because of the poison.	Snakes in general	5	
The person who has been bitten by a snake and killed it with a shot gun, will also die. The snake is to be killed only with sticks.*	Snakes in general		4
You have to wait about six months before re-entering the woods, on the contrary the poison calls again that snake which has bitten before (or other snakes).	Snakes in general	2	
If someone is urinating on a stone, the poison of the snake will go up through the urine.	Rattlesnakes		3
People carry some sticks searching for snakes at Christ's Passion. To save seven souls and their own from Hell, people have to kill seven snakes.*	Snakes in general		4
If someone kills the female, the male follows the trail to revenge its death and vice versa.	Snakes in general		7
After the snake is killed, you should examine its tail. If it is rounded in section, it means that this snake has already attacked a lot.*	Snakes in general	2	
Typically ground-dwelling poisonous snakes that are found resting on branches possess the snakestone inside them. Those snakes that usually inhabit tress never have it. This little stone serves as an amulet to repel snakes and as homemade medicine against snakebites.*	Snakes in general	14	
Black snakes feed from breastfeeding women by putting the tip of their tail in the baby's mouth so it does not cry and wake the mother. They are dangerous because the child dies from a lack of milk.	Black snakes	10	25
They posses a sting in their tails from which the poison is injected, while they bite with their mouth.	Two-headed snakes	10	2
Snake swallow their own offspring so they become more poisonous. Then they leave again from the mouth of the mother.	Snakes in general	8	1
They leave the poison on a leaf before entering the water (to take a bath and to drink). When they get out the water they take the poison (by using smell to find it).	Water snakes	4	9
They are more dangerous if one goes behind them (they attack from behind).	Rattlesnakes	2	
They even bite themselves because they are so angry.	Rattlesnakes	3	
They only have poison when they are reproducing (= laying eggs or taking care of the offspring) from June to August.	Indigo snakes Black snakes	15	
They stand due to the scissor-like stings (= tail). Then they jump (fly) to attack.	Bushmaster snakes	15	

*Beliefs that involve the direct extermination (death) of snakes.

Some snakes stand out due to their utility or because they are considered harmless to human health; even though they are culturally repellent to human sight. The species showing cultural uses are the rattlesnake, whose rattle is used as a medicinal resource, the boa, which is used as food, and the indigo snake, which serves as a biological control:

*There are people who eat even the rattlesnake. They cut it an inch from the head and another from the rattle. They eat from the middle. [...] They say it tastes like a fish! *(Mrs. C., 79 years old).

*The boa's fat is used to treat rheumatism and seizure *(Mr. B., 65 years old).

*The indigo snake eats other snakes. [...] Farmers like it *(Mr. E., 67 years old).

In the specific case of the indigo snake, because of their ophidiophagic habit, it does not seem to really bother people since it rids farmers' and ranchers' lands of the undesirable presence of other types of snakes. The apparent conservation of certain species and the indiscriminate killing of others show how ambiguous Pedra Branca's dwellers' behavior toward snakes is:

*Snakes are very ugly [...]. What beauty can they have? [...]. Just because of the poison it does not have any beauty to me [...]. Now, thinking well, the snake is not so ugly. I do not like it because of the poison and I think they are ugly [...]. It is quite clean, quite pretty [...] *(Mrs. L., 63 years old).

*As soon as we see it we get frightened, and think it is poisonous *(Mr. F., 79 years old).

*People kill it for fear of it, even though we know it is not poisonous *(F., 28 years old).

### Past and actual meanings given to the term offense

A few years ago, inhabitants from the Serra da Jibóia region used to employ the word offense only to refer to accidents with snakes. One informant said: *People did not say they were offended by scorpions. Only by snakes [...]. Now it is used with everything. Before, it was just with snakes and between people *(Mrs. L., 62 years old). Today, it has a general meaning to refer to those bites, pricks or stings caused by different animals.

A group of 34 interviewees said that when someone was stung (offended) by a snake, he/she could not say anything about the case or receive visits at home, because otherwise the effects of the envenomation would increase. Most people hide themselves to avoid being pestered. Only direct family members could visit with no harm to the victim, remaining near him/her and giving proper care (home remedies). Most people chose to not visit the victim, thus expecting their health to improve. Even so, both the relatives and the community members were also subjected to a number of restrictions, both linguistically and socially, in order to not increase the effects of the poison (its power), seriously aggravating the health of the victim. It was believed that if the words bite and sting (and their variants) were pronounced, the venom (poison in the local meaning) took even greater force at this same moment, stirring up and spreading further into the victim's body, thereby increasing the poisoning symptoms. The victim did not need to be present or listening to the conversation for that to happen. That is why people avoided talking to or near the injured person, and visits were almost completely forbidden. If someone wanted to make a visit, it was totally forbidden to speak the name of the victim (in their presence or close to them). The visitor could only go ahead and establish conversation if there was a prior expression of sympathy. All this was essential for the patient not to be frightened (with the visitor's presence and conversation), agitate the blood (the 'poison'), increase the pain, and begin to 'throw the blood out through the pores from all over the body'.

Regarding this last sentence, it is likely that such a belief comes from the fact that some people, already presenting any preexisting cutaneous lesion, when they are bitten by a young *Bothrops leucurus*, whose venom has a substance of anticoagulant action at a systemic level (different from the adult snakes), would ooze blood through these lesions [33]. For this reason, people usually perceive this leak as occurring through the pores of the whole body due to the agitation of the poison.

According to the interviewees, it was also forbidden to pronounce the word snake and the name of the species that has caused the accident. Instead, sentences or expressions such as the following were used: 'So-and-so was offended by the ground animal' (the most common and advisable expression); 'The caterpillar got me here' (spoken by the patient); 'The ant has offended him' (or other animal, but never the snake). Hence, everybody already knew that such a person had been bitten by a snake.

The folk belief concerning the fear of saying the word snake is also found in the literature. For example, in the state of Santa Catarina, south of Brazil, Cabral apud Nomura [7] recorded: 'Someone who has been bitten by a snake does not pronounce the word snake, since it would bring enormous harm to him, including death. He says he has been bitten by an animal [...]'. It is believed, then, that with these euphemisms one can avoid the bad effects of the poisoning if people have been offended by a snake, and even to ward them off.

In the village of Pedra Branca, 12 respondents commented on the charms that make it possible to visit a person bitten by a snake without causing him further harm. Examples of such charms are: the victim should drink a tea made from the chips of a doorpost from the entrance to the grocery or bar. After this procedure, a person can approach the victim without any harm (he will not bleed, for example). This tea is locally known as 'good-and-bad' because that doorpost is crossed by people with bad or good blood. Another charm is the victim wears a cross around neck, so he can receive visitors without any danger. Or the visitor brings a branch of green leaf (of any plant) and says to the victim: 'Takes that [...]. It was St. Benedict who sent it to you'.

The issue with visiting seems to be related to blood, i.e., whether or not there is compatibility between the vital fluid of the patient and the visitor's, as well as the presence and action of the snake venom in the former. Those people who were close to the victim at the exact moment he/she has been bitten could visit without fear of any harm to the offended person by their presence. They also were not requested use any charms simply because their blood was the same as that of the victim (it was not 'contrary and/or new blood'). On the other hand, a person who did not witness the accident had his/her body and blood closed. If this corporal and humoral imbalance was not counterbalanced with some charm, the difference between the two blood types during the visit would make the venom work more strongly, thus causing more pain, swelling and loss of blood through the hair. Everything seems to be a matter of matching or counterbalancing the blood to not interfere with the healing of the victim.

If the visitor was a pregnant or menstruating woman, the effect was even worse because her blood would be more closed than usual: *Pregnant women can not see the patient who has been bitten. He dies immediately *(Mr. J., 40 years old). According to local belief, which was cited by 15 participants, snakes hate pregnant women, making the poison react more strongly and with greater force and violence against the victim. Believing that the presence of pregnant or menstruating women seriously harms the victim is a widespread phenomenon throughout Brazil [7], especially with regard to not receiving any visitors. As Campos [10] says, 'the patient, to be saved, must not receive, after all, visits from pregnant women'.

### Folk beliefs related to snakes

A total of 28 beliefs related to direct or indirect accidents caused by snakes were recorded (Table 2). It is possible to see negative behavior towards snakes in all of these beliefs, including that their extermination is recommended. In many cases, fear and wrong ideas associated with these reptiles are present. Examples of some beliefs are as follows: snakes suck from the nipples of women who have had a baby; snakes leave their venom on a leaf before entering water; snakes cause chilblains; snakes have a sting in their tail from which they inject the poison; snakes can stand up thanks to the sting in the tip of the tail, thus allowing them to jump and fly up against people. Such beliefs are very common and geographically widespread, varying only by the type of snake involved and its social, cultural and environmental context [7,8,11,15,32]. According to Silva [17], some superstitions are based on the habits of snakes which are culturally interpreted and increased by imagination; others are obvious mistakes.

According to the interviewees, snakes are more poisonous from June to August because this time of the year they are mating and taking care of their offspring. It is believed that snakes become more agitated, aggressive and dangerous, increasing the potency of the venom and thus making them much more lethal:

*They are much fiercer and it is bad when they are incubating. At that time they can kill. [...]. It is in June, around St. John's day, when the offspring are born *(Mr. T., 39 years old).

*When the snake is incubating, in a few minutes the blood comes out of everywhere, the ears, the mouth, the nose. [...] June, July, and August. All snakes are laying eggs *(Mrs. N., 57 years old).

*The indigo snake is only poisonous around St. John's day. It is laying time. [...] Imagine how the others must be! *(Mr. M., 40 years old).

As seen, participants believe that birth or hatching occurs from June to August. However, this is precisely the period for reproductive activity (mating), coinciding with the coldest months of the year (in the southern hemisphere). And births usually occur from December to April, while the incidence of accidents is higher around April-June (Antonio Jorge Suzart Argôlo, pers. comm., 2008). However, whatever the season, snakes never can increase the power (quality) of their venom. No species produce it only on certain occasions, such as the birth season. As for the care of offspring, it should be said that snakes rarely exhibit parental behavior [34]; as such, they never change their behavioral state just because of the presence of young or defend them more aggressively.

### Charms, prayers and homemade remedies to prevent and treat snake bites

Until the antiophidic serum was present and available, the locals used only charms, prayers and homemade remedies to treat or protect themselves and others from snake bites. Seven charms related to the accidents caused by snakes were recorded; two of them recommend the killing of these reptiles (Table 3). Of the 13 respondents who cited the practice of putting a coral snake (preferably alive) in a bottle of brandy, 11 said they have total confidence in the effectiveness of this procedure, which becomes a traditional medicine because it is believed that one will be free from the effects of a possible bite (of any type of snake) if one drinks this liquid earlier: *It is put alive in the brandy. Then it releases the poison in it [...]. It is a remedy for any kind of snake *(Mrs. E., 61 years old). It is thought that one becomes immune to snakebites: *There was a woman who drank it and when she was offended she recovered immediately [...]. Now she can be bitten by any snake and nothing bad will happen to her *(Mrs. E., 69 years old). However, referring to the consumption of alcohol, it should be said that the alcohol initially promotes the absorption of the poison, and later, as a result of low blood pressure, slows the reaction of the body and the elimination of the toxin [7].

**Table 3:** Charms related to snakebites, according to the inhabitants of the county of Pedra Branca, Bahia State, Brazil.

Charms	Snake species	Number of individuals who	
		
		Believe	Do not believe
If the snake attacks, it has to be killed and hung in order to make the poison drops to the ground. Thus, the patient heals faster and does not hurt so much. It is worse for the patient if the snake keeps moving.*	Snakes in general	5	2
When bitten, the person has to go immediately to an anthill and eat some purple earth. If the snake comes first and eats before the person, then the victim will be weakened forever.	Rain frog snakes	8	
When bitten, the person has to go immediately to an anthill and eats some purple earth. If the snake comes first and eats before the person, then the victim gets blind and dies.	Worm lizards	6	
A whole coral snake is put inside a bottle of sugar cane brandy and this liquid is drunk to relieve the symptoms of the poisoning.*	Coral snakes	11	2
Snakes do not bite pregnant women. If you speak the name of a woman who is pregnant three times, then the snake stays quiet, waiting. Pregnant women control snakes.	Snakes in general	3	
If a pregnant woman makes a knot in her skirt, the snake becomes calm and does not bite.	Snakes in general	6	

People carry different objects in order to repel snakes.	Snakes in general	27	7

*Charms that involve the direct extermination (death) of snakes.

If the accident has been caused by a rain frog snake, it is usual to do the following charm: the victim quickly needs to eat a small amount of red earth from any anthill, before the snake that offended her does the same. If someone, by any chance, is bitten by a two-headed snake (probably *Amphisbaena *sp.), then it is believed he will be blinded and die.

The most common charm (cited by 34 respondents) and still widespread in the region is to keep objects of plant, animal or mineral origin in their wallet as amulets to make snakes flee (see Table 4), especially when walking in the woods at night. Of the individuals interviewed, 27 stated they strongly believe in the repellent and protective action of these elements; for some, it remains a daily practice carried out mainly by men (farmers, hunters etc.).

**Table 4:** Amulet-like materials carried by the inhabitants of the Serra da Jibóia region to repel snakes.

Material	Local name	N° of citation	Part used
**Plant**			
*Anacardium occidentale *L.	Cashew nut	4	Seed
*Annona crassiflora *Mart.	Aticum	7	Seed
*Bowdichia *sp.	Sucupira	4	Seed
*Joannesia princeps *Vell.	Macaw nut tree	6	Seed
**Animal**			
*Tinamus solitarius *(Vieillot, 1819)	Solitary tinamou	15	Head
*Crypturellus noctivagus zabele *(Spix, 1825)	Yellow-legged tinamou	11	Head
Venomous snakes	. . .	3	Snakestone
**Mineral**			
Arsenic bisulphur	Rosalgar	29	Stone
**Other**			
Men's trousers	Button	1	Button

'Rosalgar', or arsenic bisulphur, was cited by 29 respondents. This resource once was widely used in the studied area, but now it seems to be in disuse because its sale is prohibited. According to the interviewees, this substance was originally used as an insecticide since the powder was put on cassava leaves and inside the anthills of *Atta *spp. The magical power of this product is that it repels and/or fights snakes. The interviewees commented that, in the past, farmers put a little piece of this stone in three of the four corners of their lands. At this exact moment all snakes were made to flee through the corner without stone. However, there are side effects since if one carries the little rock at the moment that a snake attacks, the 'poison' of the stone is enhanced and the individual will be at greater risk of death. Moreover, the person should never say that he carries the stone; otherwise the substance does not have the expected effect against snakes:

*Snakes do not appear in the place when the stone is there, because the poison of the stone is greater than theirs. [...]. It only works against snakes *(Mr. T., 39 years old).

*If it is in your pocket you can walk near them, they will all go away. But there is a problem. If the snake offends you, the poison of the stone will increase the poison of the snake. It will be very difficult to escape *(Mr. M., 40 years old).

*People cannot say they have the stone or the charm is broken *(Mrs. G., 55 years old).

With respect to objects of animal origin, the interviewees mentioned two species of birds: the solitary tinamou (*Tinamus solitarius*) and the yellow-legged tinamou (*Crypturellus noctivagus zabele*). The heads of these birds are used as magical resources to keep snakes away. These species have been recorded as folk medicines in different parts of the country [35].

Except for some cases to prevent the deleterious effects of the poison (e.g., coral snake inside a bottle of brandy), the majority of the homemade medicines are recommended to treat snake bites (Table 5). Folk medicines come from plant, animal and mineral resources. Some of these folk medicines are meant to be innocuous, while others may further worsen the health of the victim, even if he is convinced of their healing powers. An example of the hazardous behavior is this use of victim's own urine and faeces to mitigate the symptoms of the snake venom while waiting for appropriate medical care.

**Table 5:** Traditional medicines cited to prevent and/or treat snakebites in the region of Serra da Jibóia, Bahia State, Brazil.

Usable resource	Local name	N° of citation	Part used	Method of use
**Plant**				
*Allium cepa *L.	Onion	6	Half cut	Put it on the bite site (it pulls the poison out)
*Allium sativum *L.	Garlic	5	Clove	Put it on the bite site (it pulls the poison out)
*Anacardium occidentale *L.	Cashew tree	12	Seed oil	Put it on the bite site (it pulls the poison out)
*Annona crassiflora *Mart.	Aticum	19	Seed	Tea made from the seeds
*Mucuna urens *D.C.	Horse-eye bean	5	Seed	Tea made from the seeds
*Joannesia princeps *Vell.	Macaw nut tree	10	Seed	Tea made from the seeds; put scraps inside a brandy bottle
*Amburana cearensis* (Fr.Allem) A.C. Smith	Amburana	8	Seed	Tea made from the seeds
*Euterpe edulis *Mart.	Juçara palm	7	. . .	. . .
*Zephyranthes *sp.	Rain lily	5	. . .	. . .
				
**Animal**				
*Tinamus solitarius*	Solitary tinamou	5	Head	Tea made from the powdered head; tied to the bite site
*Oxyrhopus *sp. *Micrurus *sp.	False coral True coral	13	Whole	Put it inside a brandy bottle and drink the liquid
*Crotalus durissus cascavella*	Rattlesnake	9	Rattle	Tea made from the powdered rattle
Poisonous snakes	. . .	14	Snakestone	Scrape it and drink it as a tea; tied it on the bite site
*Gallus gallus*	Hen	6	Egg	Eat it raw
*Homo sapiens*	Human being	7	Urine	Put it on the bite site
		14	Feaces	Tea made from the feaces
				
**Mineral**				
Arsenic bisulphur	Rosalgar	8	Stone	Tie it inside a cloth and put on the bite site
	Gasoline/kerosene	8	Liquid	Put it on the bite site
	Gunpowder	4		Tea made from the gunpowder
	Anthill soil	8	Soil around an anthill	Eat it

According to 14 interviewees, all venomous snakes (which usually inhabit the ground) that are found on branches have the so-called snakestone inside their stomach. This material of organic origin is quite powerful to treat snake bites. However, it seems that such a treatment is very difficult to do because, according to interviewees, a poisonous snake that lives on the ground is rarely seen sleeping on a branch. There are two ways to use this snakestone: to tie it on the wound, since it is believed that it will draw the poison out of the body; or scraping off a bit and taking it as a tea. It was said that this snakestone appears to already come with a hole in its center, which indicates the exact amount to be scraped off and be added into the water to prepare the tea:

Snakestone refers to a bezoar, which is actually found inside the head of some snakes and not in their intestinal tract [36]. The use of this organic substance as an antivenom is both historically ancient and geographically widespread. In India, the stone allegedly found in the head of some species is applied to the wound (bite site), stuck there without being held; then when it is soaked with as much as poison it can hold, it falls by itself. When loaded, it is put in milk to unload what it has drawn out, and then it continues to be applied until it can not stick by itself, which indicates there is no more danger to the victim [15].

Snakes are commercialized for medicinal and magic-religious purposes throughout Brazil and worldwide [35,37-39]. Unfortunately, little research has been done so far to prove the claimed clinical efficacy of snake-based products for medicinal purposes (Still apud Alves and Pereira) [9]. Additionally, the demand for live snakes or their body parts for use in traditional medicine seems to have significant implications on their populations in certain parts of the world (Fitzgerald et al. apud Alves and Pereira) [9].

Magical-religious interventions as measures to prevent and/or repel snakes were also recorded, as two women have cited two different prayers. In the folk medical oral tradition of Brazilian people, these magical-religious interventions are very common in order to get protection against venomous animals, especially snakes. The prayers are almost always directed to St. Benedict, who is the protector against snakes [7,10].

Notwithstanding, the current situation in Pedra Branca village and surroundings is that it seems there are neither linguistic nor visitation restrictions to the individual who has been bitten by a snake, and the period of recovering from such an accident has also disappeared. People do not pay attention to these things because, basically, the antivenom is now easily obtained at Santa Terezinha or Castro Alves hospitals, as one inhabitant stated: *There is the serum now. Nobody cares about it. Talking about someone who has been bitten is now allowed *(Mrs. L., 63 years old).

Over time, the use of homemade remedies, charms and prayers which were performed to prevent or treat snakebites seems to have declined. Some of the interviewed inhabitants stated that the existence and availability of the antivenom, together with the decline of snake populations in the region (mostly due to deforestation for grazing and the construction of the unpaved road within the Serra da Jibóia), have lead to the disuse (and depreciation) of local medical practices, which in older times were the only available option for healing. The following statements show the importance of traditional practices at preventing or treating snakebites, as well as some cultural changes:

*There were more charms related to snakes [...] because there were no doctors. There were a lot of homemade remedies. There's only the serum now [...]. Many people were killed by snakes. Now they are saved *(Mrs. N., 57 years old).

*There were many homemade medicines before. Now, people do not prepare them because they rightly go to the hospital and they think that folk medicine is useless [...]. They worked well before, but not now *(Mrs. E., 61 years old).

*It is very difficult to see snakes now, even in the woods, after so many fires have occurred *(Mr. E., 67 years old).

Younger generations are losing the interest in learning about these cultural practices due to sociocultural and socioeconomic changes of recent times. It is evident, among the adults in the community, that there is a sense of pessimism and complaint about this loss, as shown in the excerpts below:

*My son learns something about charms, remedies and stories because he is curious and listens to the older people [...]. The others do not care. They neither know nor understand nothing. They are interested in other things *(Mr. F., 44 years old).

*Young people from nowadays do not know anything. They are not even curious about charms and folk remedies *(Mr. J., 40 years old).

### Attitudes toward snakes and biological conservation

Snakes are often killed because inhabitants consider them as potentially dangerous and harmful to human health, thus arousing attitudes of hatefulness, aversion and fear. It is worth mentioning that some of the local beliefs and cultural practices associated with the issue of accidents by snakes already incite, clearly and directly, the killing of these reptiles.

The only recognizable example of local conservation concern for snakes in the region of Serra da Jibóia refers to indigo snakes (*Drymarchon corais*) because they prey on other snakes, including venomous ones, as well as frogs, lizards, birds and small mammals [23]. That is why farmers and ranchers seems to like and 'protect' this species because it 'cleans' their land. On the other hand, certain local beliefs (e.g., carrying objects such as amulets to repel snakes) and using several wild raw materials as folk medicines, also foster the elimination of local species selected for such purposes, which may compromise their survival in the region. In this sense, the ethnomedicinal use of *Tinamus solitarius *and *Crypturellus noctivagus zabele *deserves special attention. Zoological studies more focused on the occurrence of these species need to be done to diagnose if the local practice of using their heads as amulets, to repel snakes, has or had any effect on population densities of these two birds in the Serra da Jibóia.

The theme related to the accidents caused by venomous animals turns out to be, obviously, of great medical and sanitary interest. Any educational activity, if socially or environmentally embedded, that aims at mitigating the deleterious effects from the contact local people have with snakes, goes through an efficient public health. In this sense, the health center of the village of Pedra Branca has no informational or educational material (posters, flyers etc.) dealing with the potentially dangerous animals that occur in the region, nor does it have information or preventive measures or procedures to be followed out in the case of someone being attacked.

For this reason, it is believed that organizing workshops and disseminating informative materials in the village and surrounding communities are some of the activities that could help local residents to take a more careful (preventative) and respectful attitude towards these animals, especially those that are actually venomous. Simultaneously, environmental education based on local reality should be developed to show the ecological importance of snakes in the maintenance and balance of ecosystems. As Silva [17] points out, simply teaching, for example, that snakes should not be killed is not enough but, rather, the implications of this action to the ecosystem should be emphasized, for the production of serum etc. Such a pedagogic practice, according to the author, must 'show and give meaning to cultural diversity with regard to the representations of snakes by the community and the popular practices of prevention and treatment in cases of snake bite, so the wrong, misunderstood ideas originating from popular culture are analyzed by the community, questioned, and reflected upon by the subjects as regards their validity when they take into account the issues of snakes and snake bite [17].

Therefore, it is necessary to know how to combine the set of folk wisdom with the knowledge provided by science in order to find suitable solutions for social and environmental issues [40]. Ethnozoological information on the injuries caused by snakes and other potentially dangerous animals must be available to the community as didactic-scientific texts, written in a clear language and accompanied by illustrations. It is understood that the ethnozoological knowledge, customs and popular practices of the Serra da Jibóia inhabitants result in a valuable cultural resource which should be considered in every discussion regarding public health, sanitation and practices of traditional medicine, as well as in faunistic studies and conservation strategies for local biological diversity.

## Competing interests

The authors declare that they have no competing interests.

## Authors' contributions

DSF carried out the field research and drafted the manuscript. EMCN participated in its design and coordination, and helped to draft the manuscript. AS helped to draft the manuscript. All authors read and approved the final manuscript.
